# Age estimation via electrocardiogram from smartwatches

**DOI:** 10.1038/s44385-025-00039-5

**Published:** 2025-10-22

**Authors:** Azfar Adib, Wei-Ping Zhu, M. Omair Ahmad

**Affiliations:** Department of Electrical and Computer Engineering, Concordia University, Montreal, QC Canada

**Keywords:** Computational biology and bioinformatics, Cardiology

## Abstract

Age estimation is increasingly vital for regulating access to age-restricted services, especially to protect children online. Traditional methods—ID checks, facial recognition, and databases—raise concerns about privacy and reliability in digital contexts. Electrocardiogram (ECG) signals, reflecting heart activity, offer a promising alternative due to their age-dependent characteristics. However, prior research has largely relied on hospital-grade ECGs, limiting real-world use. To address this, we created a novel data set using smartwatch ECGs from 220 individuals across a broad age range. By testing various features and machine learning models, we achieved a mean absolute error (MAE) of 2.93 years—outperforming clinical ECG-based studies. Accuracy peaked during adolescence, when ECG changes are most pronounced. We also performed binary age classification (13–21 years), reaching 93–96% accuracy. These findings highlight smartwatch ECG’s potential for accurate and privacy-respecting age estimation.

## Introduction

Age estimation is a process that confirms users’ ages before granting access to specific age-appropriate services or products. It ensures that minors, adults, and seniors are provided with goods and services suitable for their age groups. The most common purpose of age estimation is to separate minors from adults, especially concerning age-restricted items like alcohol, tobacco, and gambling. These products are strictly regulated by laws in numerous countries to ensure they are only sold to adults. Rapid digitalization and the extensive internet connectivity worldwide have triggered a huge rise in age-dependent services in the online sphere. All major social media platforms enforce a minimum age requirement, which is typically determined by the platform itself. For example, the minimum age limit to open an account is 13 years for platforms like Facebook, Instagram, Snapchat, Twitter, and TikTok^1^. In recent times, online age estimation has become crucial to safeguard children from exposure to inappropriate online content. Different countries have implemented and reviewed laws in this regard, including the United Kingdom, Canada, United States, France^2^.

Usually, ID documents and databases are used for age estimation. However, these methods possess some risks regarding document forgery, along with significant privacy concerns. Since the COVID-19 pandemic, an exponential increase in online transaction has fueled the need of secure and privacy-preserving mechanism of online age estimation. This has created the need for anonymous age estimation schemes, which can estimate users’ age instantly from biometrics data, without knowing anything further about them. Rapid advancement of artificial intelligence during the last decade has significantly enriched biometric technologies for different purposes, including age estimation^3^.

Facial age estimation, among biometric approaches, has been the most extensively researched and successfully implemented. It has achieved commercial deployment across various sectors. State-of-the-art facial age estimation models have reported accuracy levels exceeding 95% in distinguishing minors from adults^4,5^. Recently, hand gesture-based age estimation methods have also emerged as novel biometric approaches for distinguishing between adults and minors. It leverages visual data from hand movements or poses and apply machine learning to infer age-related physiological differences^6^. Another recent technology is email-based age estimation, which determines a user’s likely age by analyzing the digital footprint associated with their email address, such as its creation date, usage history, and links to online services^7^. Researchers have also attempted to estimate human age directly through other biometrics, such as fingerprint, speech, ear, iris, etc., achieving limited success^8^.

In recent years, a coordinated international effort has accelerated the standardization of age estimation technologies, driven by the urgent need to protect children online and comply with evolving regulatory requirements. This initiative is characterized by the development of both international and national standards, engaging policymakers, industry leaders, and civil society. A significant milestone in this process is the IEEE P2089.1 standard, which offers a comprehensive framework for the design, evaluation and deployment of age assurance systems that supports both age classification and age estimation methods^9^. Since 2024, the Global Age Assurance Standards Summit has served as a key forum for aligning international standards—such as IEEE 2089.1 and the forthcoming ISO/IEC 27566-1—with local regulations to promote interoperability and best practices^10^. At the most recent summit in Amsterdam in April 2025, participants emphasized that age assurance technologies can be implemented in ways that are privacy-preserving, secure, and robust^11^.

The electrocardiogram (ECG) is a widely used diagnostic tool in cardiology that captures the electrical activity of the heart during the cardiac cycle. It is recorded as a potential difference using electrodes placed at strategic points on the body. As an inherent physiological signal and real-time indicator of liveliness, ECG is considered more secure and difficult to spoof compared to other biometric modalities.

**Fig. 1:**
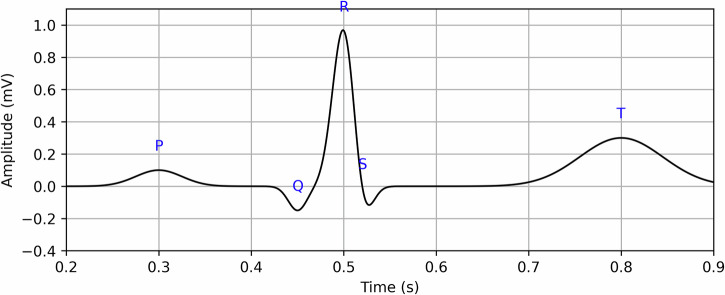
An ideal ECG waveform^16^.

Traditionally, ECG interpretation has been confined to identifying abnormalities through visual analysis of features like PR intervals, QRS complexes, and QT intervals (Fig. 1). These parameters have known age-related trends, prompting researchers to explore whether they could be reverse-mapped to estimate age. Clinical studies have established that features such as wave amplitude, duration, electrical axis, and interval variability of ECG components change with age^12–15^. A notable investigation by Macfarlane et al. analyzed ECG data across multiple ethnic groups (Caucasians, Chinese, Indians, Nigerians) and identified five age bands with marked differences in QRS interval duration: 18–29, 30–39, 40–49, 50–59, and 60+ years^16^. Early studies, such as those by Starc et al. and Ball et al., used linear regression and Bayesian models to predict cardiac age from derived ECG features. These efforts demonstrated moderate success, with R² values around 0.76, but were limited by small, homogeneous datasets and lacked adaptability across populations^17,18^.

More recent approaches utilize ECGs as holistic biometric signals, with the entire waveform analyzed using data-driven models. Deep learning architectures, particularly 1D convolutional neural networks (CNNs), have shown strong predictive capability by learning temporal and spatial dependencies directly from raw ECG traces. Attia et al. (2019) pioneered this shift, training a CNN on over 700,000 ECGs to predict chronological age, achieving a mean absolute error (MAE) of less than 7 years^19^. Other networks, such as the residual CNN proposed by Lima et al. have validated delta age (ECG age minus chronological age) as a strong predictor of all-cause and cardiovascular mortality^20^. Adib et al. extended this concept to age classification, demonstrating successful segmentation of age groups using ECG^21,22^.

One interesting application of ECG-based age estimation is cardiac diagnosis. Difference between the actual and predicted age, or delta age, has been correlated with major adverse cardiovascular events. Studies by Inojosa et al., Ladejobi et al., and Toya et al. have shown that delta age is a statistically significant predictor of cardiovascular outcomes, independent of conventional risk factors^23,24^. Inspired by these results, Strodthoff et al. tested an age-regression model on the ECG records obtained from PTB-XL database, through different combination of neural networks. They also found that testing only on healthy subjects yielded better results in each category as compared to testing only on unhealthy or all subjects^25^. In another study, Hirota et al. developed an age-prediction model based on principal component analysis, which they ran on 12,837 ECG records obtained from a cardiology specialized hospital^26^. According to their results, age estimated from 12-lead ECG showed a significant performance in mortality prediction among patients aged 60–74 years old.

While early models relied on feature analysis, newer approaches rely more on deep learning models to learn from the ECG waveform data. Despite these advancements, significant challenges remain in real-life applications of these models. Many models are trained on datasets from hospital patients, introducing bias and limiting generalizability. Additionally, black-box neural networks pose challenges in clinical interpretation. Data quality also poses an issue. ECGs often suffer from noise and inter-device variability. While some studies apply preprocessing, standard protocols are often lacking.

Till now, research on the relationship between ECG and age has largely concentrated on two key areas: (1) clinical observations on ECG in relation to age, (2) the prediction of age from ECG data using deep learning techniques. The first approach highlighted key parameters, such as heart rate variability (HRV) and QRS duration, that change with age. In the second approach, researchers focused on estimating age from ECG data using deep learning. However, several gaps remain in these approaches:

1. While deep learning has been applied by several researchers, there has been little exploration into identifying which ECG features are most representative of age.
2. All previous research has focused on predicting the exact age, with no emphasis on age classification against industry standards.
3. Additionally, these studies relied on clinical ECG data (12-lead ECG setup) obtained in hospital settings, which is cumbersome and not suitable for practical users. Availability of 1-lead ECG in smart wearables has made it more accessible recently. Smartwatch-based ECG is already being used for individual identification^27^. However, to the best of our knowledge, its exploration for age estimation is yet to be done.

To address these issues, we performed age estimation using ECG data from a smartwatch, employing feature extraction techniques and deep learning algorithms. Our contributions are summarized below:

1. Developing a dataset from smartwatch ECG data through first-hand data collection.
2. Applying different signal processing techniques for feature extraction, coupled with different deep learning methods.
3. Assessing age classification performance against industry standards.

## Results

**Fig. 2:**
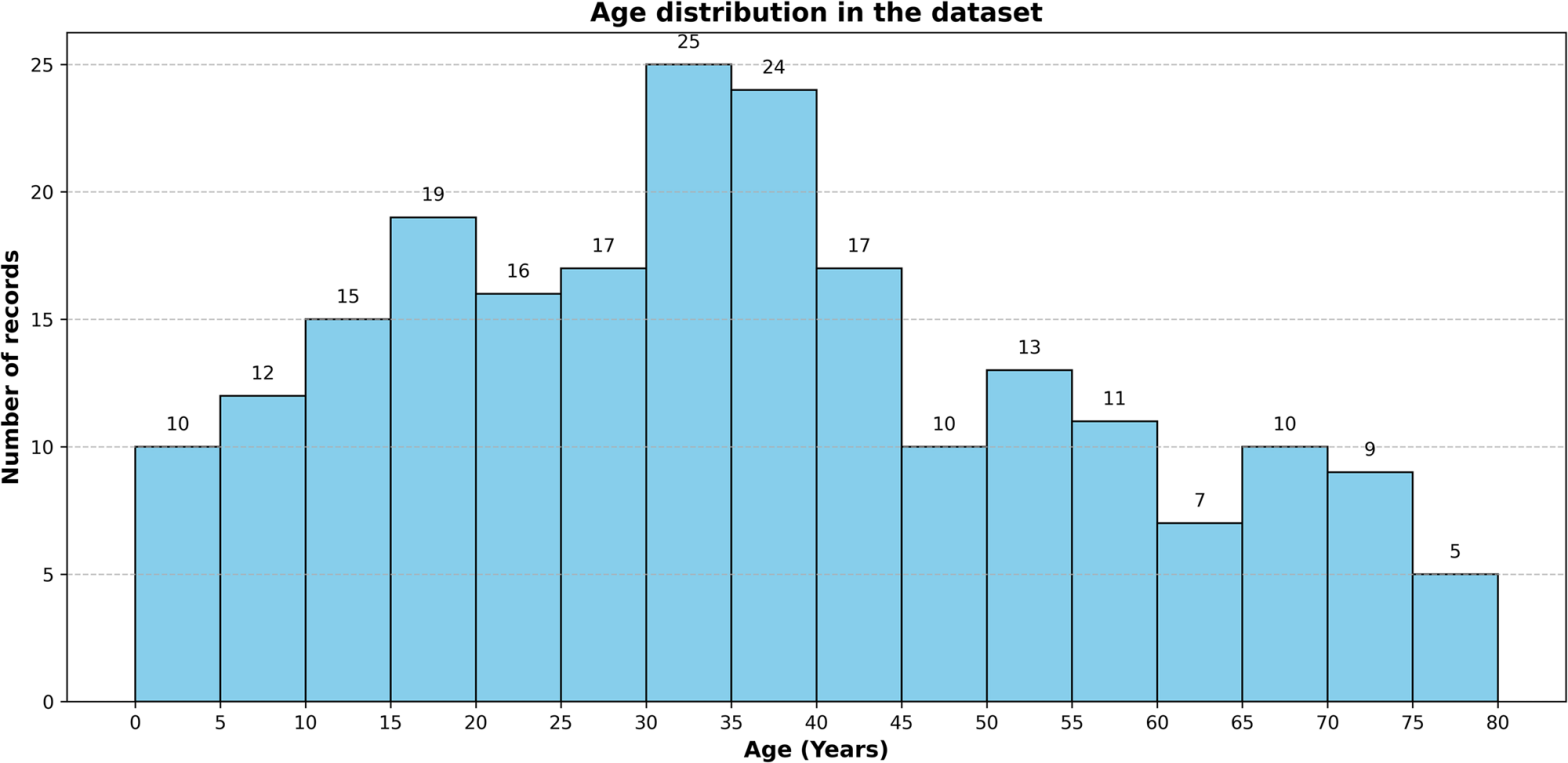
Histogram of age distribution in our dataset.

Using a FITBIT Sense smartwatch, we collected an ECG dataset comprising 220 individuals^28^. The data collection, which occurred from June 2023 to April 2025, was conducted with ethical approval from Concordia University, along with authorization from the English Montreal School Board in Montreal for data collection from minors. Each record in the dataset includes 2800 samples from a 30-s ECG recording collected at 250 Hz. Additionally, we documented each participant’s gender and cardiac health history. The dataset included individuals aged between 3 and 78 years, with a maximum falling within the 30–40-year age range. Of the 220 records, 133 were male and 87 were female. All participants, as they mentioned, were healthy with no significant history of cardiac abnormalities. Informed consent was obtained from every participant, in accordance with the ethical approval granted by Concordia University. Figure 2 displays the age distribution in our dataset.

### Age-related changes in heart rate variability (HRV)

**Fig. 3:**
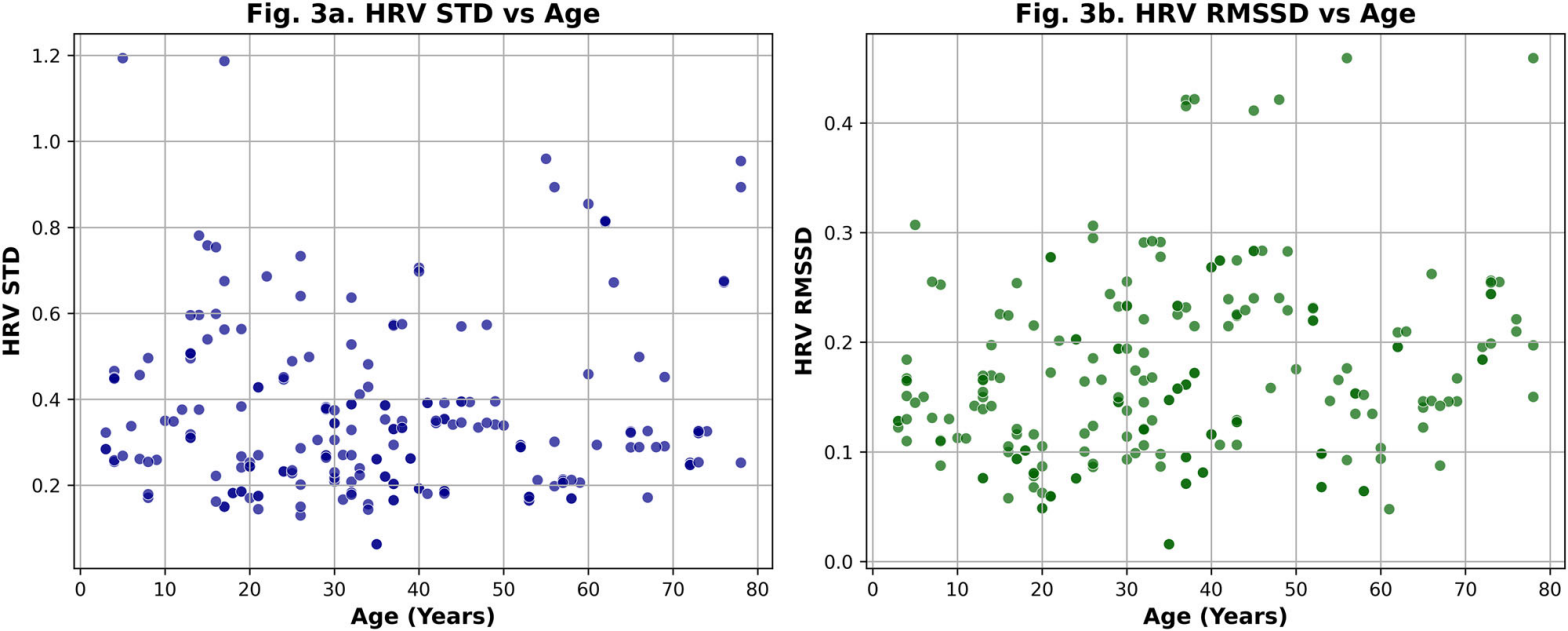
Age-related changes in Heart Rate Variability (HRV). **a** Standard deviation of heart rate variability (HRV STD) against age. **b** Root mean square of successive differences (HRV RMSSD) against age.

Figure 3a, which plots HRV STD against age and (Fig. 3b), which plots HRV RMSSD against age, reveals no definitive linear or easily interpretable pattern, despite clinical knowledge that Heart Rate Variability (HRV) generally declines with age. This discrepancy highlights a challenge: while population-level studies suggest age-related HRV trends, individual variability (e.g., lifestyle, genetics, or cardiac factors) often obscures clear patterns in real-world datasets. Traditional statistical methods or visualizations may fail to capture subtle, nonlinear relationships or interactions between variables that influence HRV. This is where neural networks can help to identify hidden association between age and ECG patterns.

### Age estimation

**Fig. 4:**
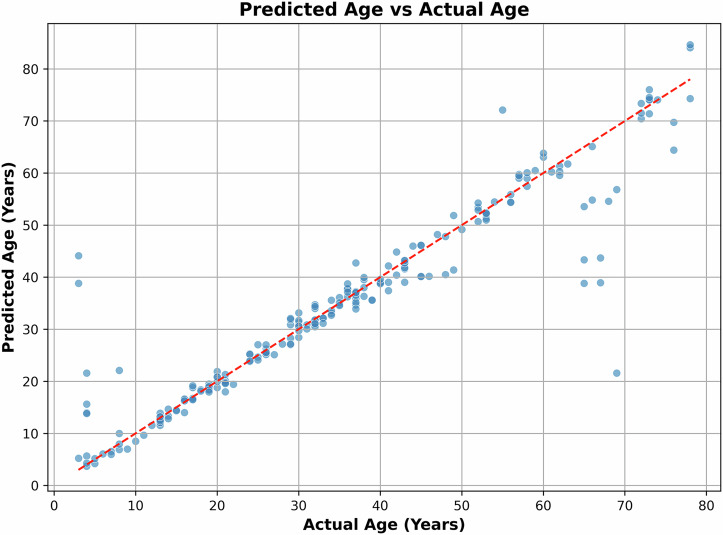
Predicted age vs. actual age, corresponding to the lowest MAE.

Table 1 compares the age estimation performance—measured by Mean Absolute Error (MAE) across various feature-model combinations. The lowest MAE of 2.93 years was achieved using a feedforward neural network applied to the de-noised ECG signal (illustrated in Fig. 4). While models like LSTM and Inception1D produced results close to this, they did not surpass it. Power Spectral Density (PSD) offered no improvement, and wavelet-based methods yielded performance comparable to the de-noised ECG. Overall, the use of extracted features did not lead to any notable performance gains across the evaluated models. These results were obtained through hyperparameter tuning on a significant scale, as depicted in Fig. 7. Table 1 presents a summary of the results obtained by using different algorithms and features.

**Table 1:** Age estimation result comparison between different methods and features

Features	Mean absolute error (MAE)			
	LSTM	Inception1D	Feedforward neural network	Transformer network
De-noised ECG	3.06	3.03	2.93	4.71
PSD	4.61	5.09	4.34	5.69
Wavelet: sym4	3.96	3.61	3.34	4.83
Wavelet:db4	3.54	3.29	3.28	4.11
Wavelet: db1 to db4	3.82	3.75	3.71	4.61
Wavelet: db1 to db10	3.15	3.47	3.21	4.33
Wavelet: sym4, db1 to db10	3.21	3.12	3.00	3.86

In recent years, different researchers have used transformer-based models for a wide range of ECG tasks, including arrhythmia classification and even age estimation. In this study, we implemented a transformer model incorporates multi-head self-attention, layer normalization, feed-forward sublayers, and residual connections, followed by global average pooling. Interestingly, this approach did not yield better results compared to our baseline model.

Moreover, the processing time for transformers was significantly higher than that for FNN, LSTM, and Inception1D. This is expected, as transformers utilize more complex architectures with self-attention and multi-head mechanisms that substantially increase computational requirements compared to the more sequential and lightweight operations in FNN and other architectures. Table 2 presents the processing times for different methods and feature sets, as measured on a Google Colab Python 3 environment with high RAM.

**Table 2:** Processing time comparison across different methods and features

Features	Processing time (seconds)			
	LSTM	Inception1D	Feedforward neural network	Transformer
De-noised ECG	37.03	44.1	22.19	79.10
PSD	46.2	52.25	31.12	108.02
Wavelet: sym4	39.32	47.2	28.8	95.21
Wavelet:db4	39.1	46.93	29.03	95.44
Wavelet: db1 to db4	40.3	47.03	30.16	104.2
Wavelet: db1 to db10	40.7	47.11	30.22	111.19
Wavelet: sym4, db1 to db10	42.3	47.21	30.97	120.02

Transformers, with their self-attention mechanisms, usually outperform both CNNs and LSTMs in modeling long-range dependencies and capturing global signal context without succumbing to vanishing gradients. Attention mechanisms enable models to focus selectively on the most informative regions of ECG tracings, improving interpretability and predictive performance—especially as ECG datasets become larger. Nevertheless, transformer and attention-based methods typically demand more computational resources, larger datasets, and careful architectural design to avoid overfitting. In smaller-scale or resource-constrained scenarios with limited training data (as in this case: 220 subjects, single-lead smartwatch ECG), the relative simplicity and data efficiency of FNN architectures can be advantageous and pragmatic.

Table 3 presents the age estimation performance across different age segments, while using a feedforward neural network on de-noised ECG. Notably, the results were poorer for the 1–5 and 66– 70-year groups, while the best performance was observed in the 11–15 and 16–20-year segments. The table also includes the total number of records (training and test data) for each segment to assess whether limited data size influenced performance. However, data scarcity does not appear to be a significant factor affecting the results. This trend aligns with clinical observations of how ECG characteristics change with age^12–16^. Our hearts and nervous systems undergo significant changes in early adulthood, making these differences easier to detect. As we age, our heart rate variability (HRV) decreases rapidly at first, then more slowly, making it harder to pinpoint exact ages in older groups. It is important to note that while HRV decreases with age, resting heart rate itself tends to remain relatively stable or decrease only slightly in healthy adults. The more pronounced changes in HRV and other ECG features in younger adults contribute to more accurate age estimation for this group compared to older individuals. This result is further illustrated in Fig. 4, which plots actual age against predicted age. Ideally, in the best-case scenario, the points should align closely with the 45° line, indicating perfect prediction. While the predictions remained consistent for most age groups, noticeable deviations were observed in the 1–10 and 60–70-year ranges.

**Table 3:** Age estimation results for different age segments using a feedforward neural network on the de-noised ECG

Age segment (years)	No. of total records	MAE	SDAE	r
1–5	14	16.84	15.87	−0.56
6–10	10	2.85	3.84	0.60
11–15	19	0.84	0.43	0.85
16–20	24	0.86	0.46	0.82
21–25	14	1.18	0.86	0.57
26–30	17	1.05	0.89	0.72
31–35	25	1.10	0.64	0.84
36–40	22	1.54	1.69	0.62
41–45	15	1.09	0.75	0.80
46–50	6	2.45	1.24	0.26
51–55	16	3.48	1.09	0.77
56–60	10	1.79	2.73	−0.27
61–65	9	1.15	1.17	−0.49
66–70	5	15.30	11.04	−0.24
71–75	11	1.22	0.48	−0.08
76–80	3	6.25	4.74	0.90
Total	220	2.93	6.2	0.94

**Fig. 5:**
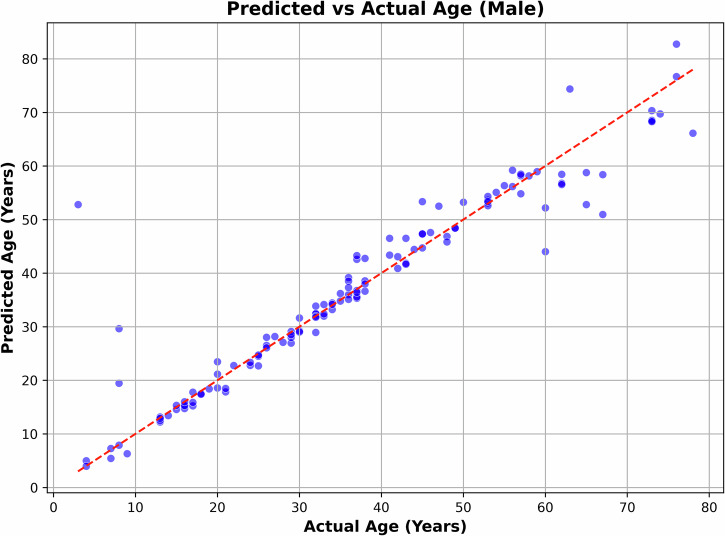
Predicted age vs. actual age, for male participants.

**Fig. 6:**
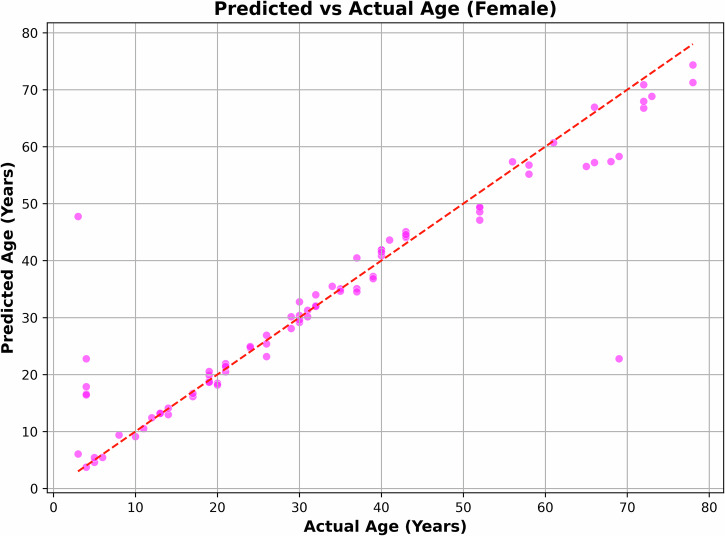
Predicted age vs. actual age, for female participants.

In our overall dataset, 60.5% of participants were male (133 individuals) and 39.5% were female (87 individuals). For male participants, the model achieved a Mean Absolute Error (MAE) of 2.85. For female participants, the MAE was 3.06. Figures 5, 6 demonstrate the plots of actual age against predicted age for male and female participants.

**Fig. 7:**
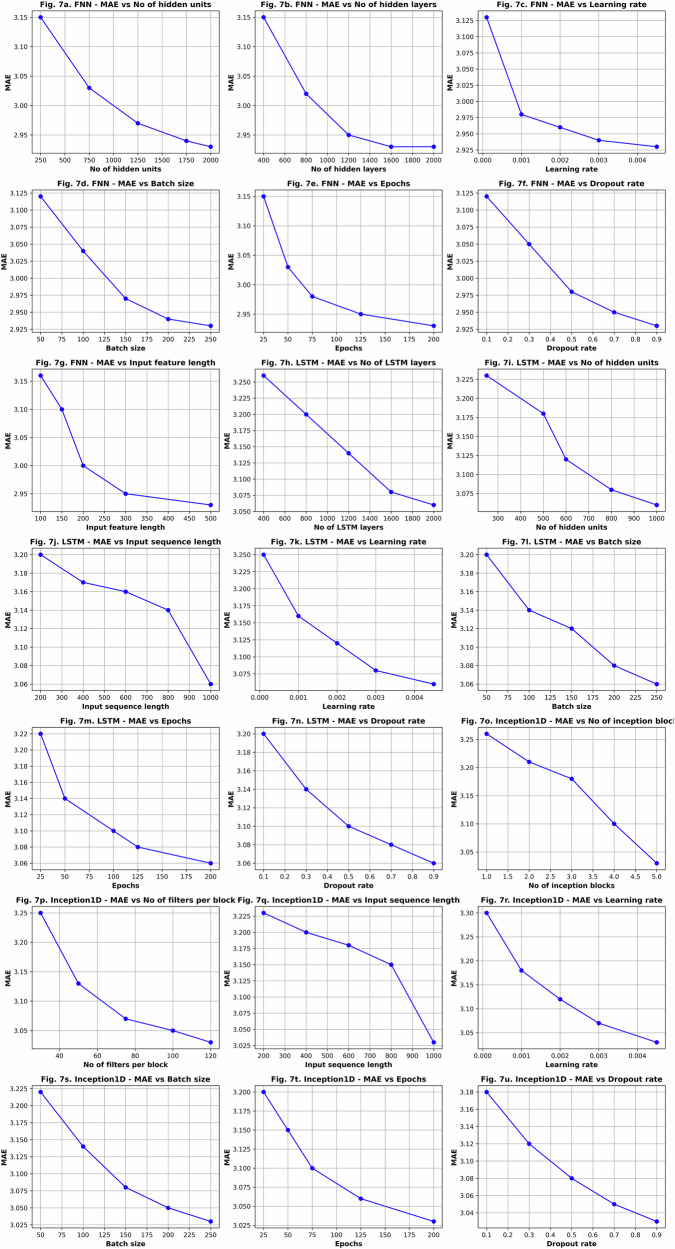
Hyperparameter optimization for age estimation using FNN, LSTM, and Inception-1D models. FNN: MAE vs **a** number of hidden units, **b** Number of hidden layers, **c** learning rate, **d** batch size, **e** epochs, **f** dropout rate, **g** input feature length. LSTM: MAE vs **h** number of LSTM layers, **i** number of hidden units, **j** input sequence length, **k** learning rate, **l** batch size, **m** epochs, **n** dropout rate. Inception-1D: MAE vs **o** number of inception blocks, **p** number of filters per block, **q** input sequence length, **r** learning rate, **s** batch size, **t** Epochs, **u** dropout rate.

The best MAE value of 2.93 from the Feedforward Neural Network was obtained in these hyperparameter settings: 2000 hidden layers and hidden units, learning rate of 0.005, batch size of 250, 200 epochs and a dropout rate of 0.9. Figure 7 illustrates the optimization of hyperparameters for age estimation from denoised ECG signals using FNN, LSTM, and Inception-1D models.

**Fig. 8:**
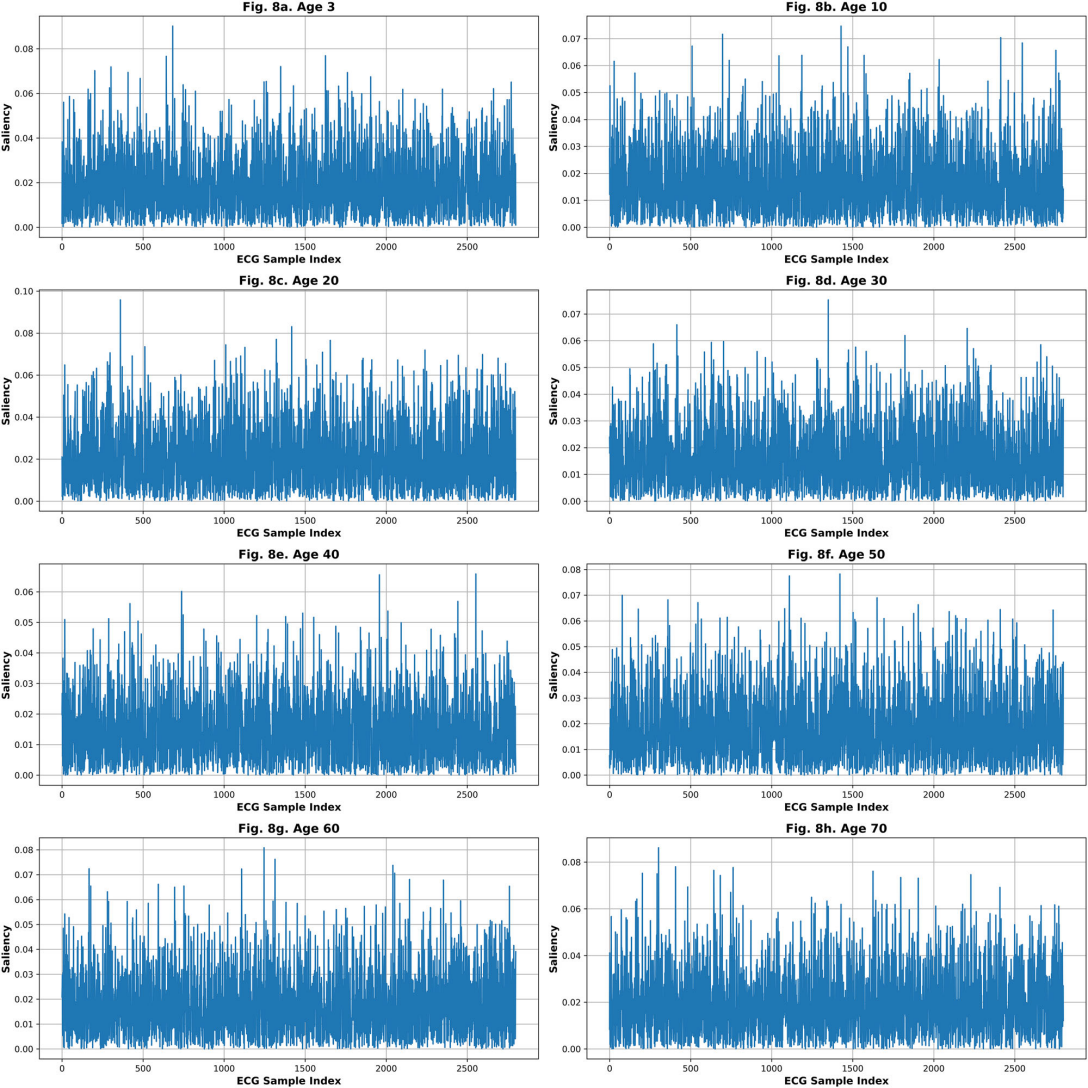
Saliency map revealing key ECG features for age prediction from de-noised ECG using FNN in different ages. **a** age 3, **b** age 10, **c** age 20, **d** age 30, **e** age 40, **f** age 50, **g** age 60, **h** age 70.

Figure 8 illustrates saliency maps derived from a feedforward neural network, highlighting the most influential features contributing to age prediction from denoised ECG waveforms for samples representing eight distinct ages (3, 10, 20, 30, 40, 50, 60, and 70 years). Interestingly, the saliency patterns closely resemble the morphology of typical ECG signals, with pronounced emphasis on the QRS complex and R-peaks. This alignment is particularly compelling, as it demonstrates that the model’s predictive focus corresponds with well-established physiological markers.

### Age classification

To address real-life needs, we performed binary classification within specific age ranges on the same dataset, using the mentioned models and features, based on age thresholds of 12 and 21 years. This method aims to classify whether the age output falls below or above these thresholds. The results are presented in Tables 4–6. These results were consistent across all models, which indicates the potential for this scheme to segregate adults and minors in accordance with real life needs.

**Table 4:** Results of age classification using a feedforward neural network on the de-noised ECG

Age	Acc	CI	TPR	TNR	FPR	FNR	PPV	NPV	F1
13	0.93	(0.85,1.00)	0.96	0.65	0.35	0.04	0.96	0.68	0.96
14	0.95	(0.89,1.00)	0.98	0.77	0.23	0.02	0.96	0.89	0.97
15	0.95	(0.89,1.00)	0.98	0.76	0.24	0.02	0.96	0.90	0.97
16	0.96	(0.90,1.00)	0.99	0.81	0.19	0.01	0.96	0.94	0.98
17	0.96	(0.90,1.00)	0.99	0.83	0.17	0.01	0.96	0.97	0.98
18	0.95	(0.89,1.00)	0.99	0.80	0.20	0.01	0.95	0.95	0.97
19	0.94	(0.87,1.00)	0.96	0.85	0.15	0.04	0.96	0.87	0.96
20	0.97	(0.92,1.00)	0.99	0.91	0.09	0.01	0.97	0.96	0.98
21	0.96	(0.90,1.00)	0.98	0.90	0.10	0.02	0.96	0.95	0.97

**Table 5:** Results of age classification using LSTM on the de-noised ECG

Age	Acc	CI	TPR	TNR	FPR	FNR	PPV	NPV	F1
13	0.94	(0.87,1.00)	0.97	0.65	0.35	0.03	0.96	0.75	0.97
14	0.95	(0.89,1.00)	0.99	0.74	0.26	0.01	0.96	0.92	0.97
15	0.96	(0.90,1.00)	1.00	0.76	0.24	0.00	0.96	1.00	0.98
16	0.95	(0.89,1.00)	0.98	0.78	0.22	0.02	0.96	0.9	0.97
17	0.94	(0.87,1.00)	0.98	0.78	0.22	0.02	0.95	0.91	0.97
18	0.94	(0.87,1.00)	0.98	0.82	0.18	0.02	0.95	0.90	0.97
19	0.94	(0.87,1.00)	0.96	0.87	0.13	0.04	0.96	0.87	0.96
20	0.95	(0.89,1.00)	0.96	0.91	0.09	0.04	0.97	0.89	0.97
21	0.94	(0.87,1.00)	0.96	0.90	0.10	0.04	0.96	0.90	0.96

**Table 6:** Results of age classification using Inception1d on the de-noised ECG

Age	Acc	CI	TPR	TNR	FPR	FNR	PPV	NPV	F1
13	0.93	(0.85,1.00)	0.95	0.74	0.26	0.05	0.97	0.65	0.96
14	0.96	(0.90,1.00)	0.99	0.81	0.19	0.01	0.97	0.93	0.98
15	0.96	(0.90,1.00)	0.98	0.85	0.15	0.02	0.97	0.91	0.99
16	0.96	(0.90,1.00)	0.98	0.86	0.14	0.02	0.97	0.91	0.98
17	0.96	(0.90,1.00)	0.97	0.9	0.10	0.03	0.98	0.88	0.97
18	0.96	(0.90,1.00)	0.98	0.89	0.11	0.02	0.97	0.99	0.97
19	0.96	(0.90,1.00)	0.97	0.91	0.09	0.03	0.98	0.90	0.97
20	0.96	(0.90,1.00)	0.96	0.94	0.06	0.04	0.98	0.89	0.97
21	0.96	(0.90,1.00)	0.97	0.93	0.07	0.03	0.97	0.92	0.97

Table 7 presents a comparison of our age-estimation results with two recent studies conducted using clinical ECG data. Our study, based on smartwatch ECG signals, achieved significantly better performance. However, a limitation of our approach is that our dataset included only healthy individuals, whereas the other studies considered both healthy and non-healthy participants.

**Table 7:** Age estimation result comparison with other similar studies

Study	Data type	Methodology	MAE
Attia et al.^19^	Primary: clinical ECG	CNN	≥7.0 (non-healthy) <7.0 (healthy)
Strodthoff et al.^25^	Secondary: PTB-XL database	Inception, RESNET, LSTM	7.12 (all) 6.80 (healthy) 7.37 (non-healthy)
Lima et al.^20^	Primary: clinical ECG	CNN	≥8.0 (non-healthy) <8.0 (healthy)
Our method	Primary: smartwatch ECG	Feedforward neural network	2.93 (healthy)

Table 8 presents a comparative analysis of age estimation performance between our method and an existing facial recognition-based age estimation solution^29^. Our ECG-based age estimation achieves a mean absolute error (MAE) of 2.93 years compared to 2.5 years for facial recognition methods.

**Table 8:** Age estimation result comparison with other method

Metric	ECG-based age estimation (this Study)	Facial recognition-based age estimation^29^
Accuracy (MAE)	2.93 years (age range: 3–78 years)	2.5 years (age range: 6–70 years)
Skin tone dependency	None observed	Performance varies with skin tone
Health condition impact	Significant, according to prior studies.	None reported
Gender variation	Minimal	Minimal
Anonymization	Ensures anonymity through biomedical data processing.	Not fully anonymous; privacy concerns exist due to image data.

Unlike facial recognition, ECG-based estimation shows no dependency on skin tone and supports privacy through on-device processing. Most importantly, it facilitates anonymized data handling. However, its accuracy can be affected by cardiovascular conditions, while facial recognition methods report no such health-related impact. While facial recognition often achieves high accuracy, it raises significant privacy concerns and can be susceptible to spoofing, lighting conditions, and demographic biases.

In contrast, ECG-based methods offer inherent liveness detection, are less intrusive, and are difficult to replicate or forge. Both approaches show minimal variation in performance across genders. In contrast, facial age estimation has been extensively validated across large-scale datasets, largely due to the wide availability of facial images. ECG-based age estimation, by comparison, is still an emerging field. In this study, our experiments were conducted on a limited number of samples. However, with broader access to ECG data—particularly from popular smartwatch manufacturers, this approach could be further validated and refined at scale, potentially offering a robust and privacy-preserving alternative for age estimation.

## Discussion

The Fitbit Sense Smartwatch is equipped with a low-power custom ARM Cortex processor designed primarily for energy-efficient health monitoring rather than high-performance computation. Its on-device processing capabilities are sufficient for basic real-time tasks such as heart rate variability (HRV) calculations and ECG signal filtering. However, more complex analyses, including arrhythmia detection or age prediction from ECG, are typically offloaded to a paired smartphone or cloud-based systems due to the device’s limited computational resources. Latency for simple operations remains low (around 10–100 ms), enabling a smooth user experience for basic monitoring tasks. From a computational cost perspective, Fitbit Sense performs lightweight processing on ECG signals, such as noise reduction and compression. These preprocessing steps help reduce data size and transmission load, but the device does not handle resource-intensive operations like deep learning inference or statistical modeling. Those functions, when supported by Fitbit’s ecosystem, are executed off-device, preserving the wearable’s processing and memory capacity.

In terms of battery usage, collecting ECG data has a relatively small but noticeable impact. A single ECG session, which typically lasts between 30 s and 2 min, uses a moderate amount of power primarily due to sensor activation and continuous Bluetooth transmission. While each session consumes only about 1–2% of the battery, regular use can shorten the device’s average battery life from around 6 days to approximately 3–4 days depending on frequency and other concurrent sensor usage.^30^. Regarding data security and anonymization, Fitbit uses Bluetooth Low Energy (BLE) protocols with encryption to securely transmit ECG data to the user’s paired smartphone. Once uploaded, the data is stored in the Fitbit cloud, now operated by Google, where it is protected through end-to-end encryption. For research and analytics purposes, Fitbit de-identifies health data in compliance with regulations like the General Data Protection Regulation (GDPR) and the Health Insurance Portability and Accountability Act (HIPAA). This means that any personally identifiable information (PII) is stripped from datasets before analysis or third-party sharing. ECG data collection is initiated manually by the user, typically by touching the metal frame during a guided session, reducing the risk of unintended background data capture. Users maintain control over their health data through options to export or delete their records via the Fitbit dashboard. Fitbit’s privacy policies also allow users to make data residency and deletion requests in accordance with regional privacy laws such as the GDPR and the California Consumer Privacy Act (CCPA)^31^.

The transferability of our ECG-based machine learning model—from data acquired using the Fitbit Sense to other consumer-grade wearable devices—is influenced by multiple hardware and signal processing factors, though not all have equal impact. Electrode configuration and signal quality tend to be less critical barriers, especially for wrist-worn wearable applications. Most consumer wearables use similar single-lead, wrist-based setups, which means ECG morphology remains broadly comparable across devices. Variations in signal quality due to skin contact or minor hardware noise can typically be addressed through standard preprocessing techniques for artifact removal. However, substantial differences—such as a shift from wrist to chest-based electrode placement or significantly increased noise—can affect model generalizability unless addressed through retraining.

In contrast, the sampling rate is a more significant constraint. A model trained on Fitbit Sense data may lose accuracy and introduce bias when applied to signals captured at lower sampling rates, as certain waveform features may be lost or distorted. While higher sampling rates offer richer data, they can also introduce additional noise and increase the demand for more advanced filtering. Therefore, frequency filtering and signal preprocessing must be carefully tailored to the specific sampling characteristics of each device^32–34^. Table 9 contains a summary of these factors.

**Table 9:** Key factors affecting model transferablity across ECG monitoring devices

Factor	Impact on Model Transferability
Sampling rate	Alters signal resolution and information content; hence, the model needs to be carefully adapted.
Electrode configuration	For any single-lead ECG device, ECG morphology remains broadly comparable, with some minor adjustment in the model.
Signal noise	Affects signal’s reliability; can be mitigated through preprocessing without any major change in the model.

Modern wearable devices such as smartwatches and fitness trackers have steadily advanced in computational capability, making on-device inference for biomedical signals like ECG both feasible and highly relevant for real-world deployment. Although the present study relies on off-device analysis, lightweight models optimized for on-device execution would substantially enhance its practical applicability and future scope. To enable real-time, privacy-preserving age estimation directly on wearables, model compression techniques—including pruning, quantization, and knowledge distillation—provide promising pathways. Pruning removes redundant parameters, lowering computational and memory demands with minimal impact on accuracy. Quantization reduces model weights and activations to lower-precision formats, thereby decreasing model size and enabling efficient execution on device-specific hardware^35^. Collectively, these approaches have already demonstrated the ability to support real-time ECG algorithms on commercial wearable platforms. We anticipate that applying these strategies to our models will enable seamless transition from research-grade, off-device inference to end-to-end wearable solutions in the future.

Developing an accurate, secure, easily deployable, and anonymous biometric-based age estimation system remains a significant challenge. Despite certain limitations, the electrocardiogram (ECG) shows considerable promise, especially when integrated into accessible platforms such as smartwatch-based ECG applications. To the best of our knowledge, this study is among the first to explore age estimation using ECG signals collected from smartwatches. Although our data set was limited in size and included only healthy subjects, the promising results for both age estimation and age classification highlight the potential for further research in this area. We compared here the effectiveness of various features and machine learning algorithms to identify an optimal approach. In addition to age estimation, we also performed age classification to address practical usage scenarios. Interestingly, a simplified model using the original denoised ECG signal outperformed more complex, feature-based models. This is likely due to the data set comprising only healthy individuals. While this approach may seem technically less sophisticated, it offers significant advantages for real-world applications, enabling easier implementation and faster processing on devices like smart wearables. Overall, the encouraging results from this study provide a strong foundation for future experimentation and the potential real-world deployment of ECG-based age estimation and classification technology.

## Methods

In accordance with standard procedures, this research obtained a Certification of Ethical Acceptability for Research Involving Human Subjects from the Concordia University Human Research Ethics Committee. The certification is valid for one year and was initially granted in June 2023, with subsequent renewals in 2024 and 2025. As part of the ethical requirements, the corresponding author also completed the Tri-Council Policy Statement: Course on Research Ethics (TCPS 2) mandated by the Government of Canada. The research methodology consisted of the following steps:

1. De-noising: The major sources of noise in ECG signals include baseline wander, power line interference, and motion artifacts^13^. To address this, we applied a 4th-order Butterworth bandpass filter on the raw ECG data for de-noising. The frequency range was adjusted based on experimental requirements, with the optimal filtering found to be in the range of 1–40 Hz.
2. Heart Rate Variability (HRV) Analysis: We plotted HRV STD (HRV Standard Deviation, also known as SDNN) and HRV RMSSD (HRV root mean square of successive differences)*—*for the entire dataset, against age to better understand how heart rate variability (HRV) changes as people grow older. These two metrics are widely used. HRV STD reflects overall variability in heart rate while HRV RMSSD specifically measures short-term, beat-to-beat variability^15^.
3. Feature extraction: Previous studies have performed age estimation using only ECG. In our study, we analyzed the ECG alongside different extracted features. To improve the interpretability of our algorithm, we used various features:
  i. Discrete Wavelet Transform (DWT): DWT previously yielded promising results in age estimation from ECG in our earlier research^21,22^. DWT decomposes a signal into different levels of approximation (low-frequency components) and detail (high-frequency components) by passing it through a series of filters. The output is a set of coefficients representing the signal’s detail and approximation at each level of decomposition. DWT has long been a versatile tool in ECG analysis, widely applied for tasks such as denoising and arrhythmia detection. In our scheme, we employed ten different mother wavelets from the Daubechies family (db1 to db10) and one from the Symlet family (sym4). All the approximation and detailed coefficients were organized into a matrix, where each row represents the coefficients, providing a comprehensive representation of the signal’s frequency content at multiple resolutions. This approach was made considering the factors below:
  - Signal morphology matching: each wavelet family has distinct shapes that may better match certain ECG waveform features. The range from db1 to db10 offers different levels of smoothness, allowing for the capture of both sharp transitions (like QRS complexes) and smoother components (like P and T waves) in ECG signals.
  - Robustness: a diverse set of features extracted using different wavelets can make the algorithm more robust to individual variations and noise. Each wavelet may highlight different aspects of the ECG signal.
  - Frequency localization: different wavelets provide varying degrees of frequency localization, which is crucial for analyzing the spectral content of ECG signals across different scales. This is more evident as heartbeat and corresponding frequencies of ECG vary with age.
  ii. Power spectral density: in ECG analysis, PSD is commonly used to study the frequency content of heart rate variability (HRV) signals, which reflect the autonomic nervous system’s activity. For age analysis, PSD is particularly useful because it can reveal age-related changes in cardiovascular physiology. Different frequency bands in the PSD (e.g., low-frequency and high-frequency components) correspond to specific biological processes, such as parasympathetic and sympathetic nervous system regulation. Ageing tends to alter the balance between these frequency components, making PSD a valuable tool for extracting features that correlate with age^36^.
4. Deep learning: we used the following schemes-

i. LSTM (Long Short-Term Memory): LSTMs are a type of recurrent neural network (RNN) designed to capture long-term dependencies in sequential data. They are highly effective for ECG because they can model temporal patterns and subtle variations in heart rhythms.
ii. ii.Inception1D: Inception1D is tailored for time-series feature extraction, using parallel convolutional layers with different kernel sizes to capture patterns at multiple temporal resolutions. It can detect both short-term and long-term features in heartbeats, such as QRS complexes and P-T wave variations.
iii. Feedforward neural network: we used a fully connected feedforward neural network designed for a regression task. It begins with an input layer that accepts features shaped according to the number of input variables in the dataset. The first hidden layer consists of 256 neurons with a ReLU activation function, followed by a dropout layer with a rate of 0.1 to reduce overfitting. The second hidden layer has 128 neurons, also using ReLU activation, and is followed by another dropout layer with the same rate. Finally, the output layer is a single neuron without an activation function, making it suitable for predicting a continuous target variable. This architecture provides a balance between learning capacity and regularization.
iv. Transformer Network: We used a transformer network comprising stacked layers of multi-head self-attention mechanisms for contextual feature extraction across the full input sequence^37^. Multi-head attention enables the model to jointly capture dependencies from multiple subspaces, thereby improving representation diversity, while position-wise feed-forward networks applied to each token embedding enhance non-linear transformation capacity. Each sublayer is coupled with residual skip connections and layer normalization to promote stable gradient propagation and accelerate convergence. This architectural design facilitates efficient modeling of both local and long-range dependencies without recurrence or convolution. To derive a fixed-length representation compatible with downstream classification, we employed global average pooling over token embeddings, yielding a compact yet semantically enriched vector summarizing the sequence information^38^.

In all the scenarios, 60% of the data was used for training, 20% used for validation and 20% were used for testing.

5. Performance metrics: in line with the guidelines established, we used the matrices below to evaluate performance for age estimation^39^.

Mean absolute error (MAE):

1 $$\mathrm{MAE}=\frac{1}{{\rm{n}}}\mathop{\sum }\limits_{{\rm{i}}=1}^{{\rm{n}}}|{{\rm{p}}}_{{\rm{i}}}-{{\rm{a}}}_{{\rm{i}}}|$$

Standard deviation (SD):

2 $$\sqrt{{\mathrm{SD}}_{\mathrm{AE}}=\frac{1}{{\rm{n}}-1}\mathop{\sum }\limits_{{\rm{i}}=1}^{{\rm{n}}}{\left({\mathrm{AE}}_{{\rm{i}}}-\mathrm{MAE}\right)}^{2}}$$

Pearson’s correlation coefficient (r):

3 $${\rm{r}}=\frac{n\sum {{{\rm{a}}}_{{\rm{i}}}{\rm{p}}}_{{\rm{i}}}-\sum {{\rm{a}}}_{{\rm{i}}}\sum {{\rm{p}}}_{{\rm{i}}}\,}{\sqrt{n}(\sum {{\rm{p}}}_{{\rm{i}}}2-\left(\sum {{\rm{p}}}_{{\rm{i}}})2\right).\,\sqrt{n}(\sum {{\rm{a}}}_{{\rm{i}}}2-(\sum {{\rm{a}}}_{{\rm{i}}})2)}$$

where, *n* = Number of samples ${{\rm{p}}}_{{\rm{i}}}$ = Predicted age${{\rm{a}}}_{{\rm{i}}}$ = Actual age${{\rm{AE}}}_{{\rm{i}}}$ = $|{{\rm{p}}}_{{\rm{i}}}-{{\rm{a}}}_{{\rm{i}}}|$ = Absolute error for the *i*-th sample

Confidence Interval: We used the below formula for estimating 95% confidence interval around the thresholds of age classification:

4 $$\mathrm{Confidence\; Interval},\mathrm{CI}=\left({\rm{p}}-{\rm{Z}}\sqrt{\frac{{\rm{p}}\left(1-{\rm{p}}\right)}{{\rm{n}}}},{\rm{p}}+{\rm{Z}}\sqrt{\frac{{\rm{p}}\left(1-{\rm{p}}\right)}{{\rm{n}}}}\right)$$

where, *n* = Number of samples${\rm{p}}$ = Classification accuracyZ = 1.96 (the Z-score corresponding to a 95% confidence level)

For age classification, we used the following metrics (described in Tables 10, 11) for performance evaluation in accordance with existing industry standards:

**Table 10:** Performance metrics for age classification

Abbreviation	Full form	Formula
TPR	True Positive Rate (Sensitivity)	TP/(TP + FN)
TNR	True Negative Rate (Specificity)	TN/(FP + TN)
FPR	False Positive Rate (Fall-out)	FP/(FP + TN)
FNR	False Negative Rate (Miss rate)	FN/(FP + TN)
PPV	Positive Predictive Value	TP/(TP + FP)
NPV	Negative Predictive Value	TN/(TN + FN)
F1-score		(2*PPV*TPR)/(TPR + PPV)
Accuracy		(TP + TN)/(TP + TN + FP + FN)

**Table 11:** Basic parameters for the performance metrics

Actual age	Predicted age	
	Above threshold	Below threshold
Above threshold	True positive (TP)	False negative (FN)
Below threshold	False positive (FP)	True negative (TN)

These age thresholds were selected for age classification performance measurement due to their significance in real-world regulatory and social contexts. Globally, the age of 13 is widely recognized as the threshold for digital consent and marks the transition from early childhood to adolescence, especially in online privacy regulations such as the Children’s Online Privacy Protection Act (COPPA). The age of 18 is commonly viewed as the legal age of majority, granting individuals full legal rights and responsibilities in many jurisdictions. Similarly, 21 is often associated with access to age-restricted services such as alcohol, gambling, and certain online platforms. These thresholds represent critical decision points for content access, parental consent, and user eligibility.

## Data Availability

The authors declare that the data supporting the findings of this study are available publicly at this GitHub repository: https://github.com/azfaradib/smartwatch-ecg-age-prediction.

## Code availability

The authors declare that the code used in this research are available publicly at the Github repository: https://github.com/azfaradib/smartwatch-ecg-age-prediction. The Python scripts are compatible with Python 3.10, and the MATLAB code can be executed using MATLAB R2024a or later versions.
